# Assessment of Seasonal Variability in Phosphorus Biodynamics by Cosmogenic Isotopes ^32^P, ^33^P around Balaklava Coast

**DOI:** 10.3390/ma16051791

**Published:** 2023-02-22

**Authors:** Mariya A. Frolova, Nikolay A. Bezhin, Evgeniy V. Slizchenko, Ol’ga N. Kozlovskaia, Ivan G. Tananaev

**Affiliations:** 1 Department of Chemistry and Chemical Engineering, Sevastopol State University, Universitetskaya Str., 33, 299053 Sevastopol, Russia; 2 Radiochemistry Laboratory, Vernadsky Institute of Geochemistry and Analytical Chemistry, Russian Academy of Sciences (GEOKHI RAS), Kosygin St., 19, 119991 Moscow, Russia; 3 Department of Nuclear Technology, Far Eastern Federal University, Sukhanov Str., 8, 690091 Vladivostok, Russia

**Keywords:** sorbents, PAN-Fe(OH)_3_, phosphorus, ^32^P, ^33^P, phosphorus biodynamics, Balaklava coast

## Abstract

The sorption efficiency of phosphorus from seawater by aluminum oxide and sorbents based on Fe(OH)_3_ obtained by various methods (using prepared sodium ferrate or precipitation of Fe(OH)_3_ with ammonia) was assessed. It was shown that phosphorus was recovered most efficiently at a seawater flow rate of one-to-four column volumes per minute with a sorbent based on hydrolyzed polyacrylonitrile fiber with a precipitation of Fe(OH)_3_ with ammonia. Based on the results obtained, a method for phosphorus isotopes recovery with this sorbent was suggested. Using this method, the seasonal variability of phosphorus biodynamics in the Balaklava coastal area was estimated. For this purpose, the short-lived isotopes of cosmogenic origin ^32^P and ^33^P were used. Volumetric activity profiles of ^32^P and ^33^P in particulate and dissolved forms were obtained. Based on ^32^P and ^33^P volumetric activity, indicators of phosphorus biodynamics were calculated: the time, rate, and degree of phosphorus circulation to inorganic and particulate organic forms. In spring and summer, elevated values of phosphorus biodynamic parameters were determined. This is explained by the peculiarity of the economic and resort activities of Balaklava, which negatively affect the state of the marine ecosystem. The results obtained can be used to assess the dynamics of changes in the content of forms of dissolved and suspended phosphorus, and the biodynamic parameters when performing a comprehensive environmental assessment of the state of coastal waters.

## 1. Introduction

All manifestations of life on Earth are based on the processes of the exchange of chemical elements between organisms and the environment [1]. The consumption of nutrients by marine microorganisms can lead to a deficit of one or more elements in the marine environment. In such cases, according to Liebig’s principle [2], the element with the highest deficit determines the biological productivity of the ecosystem. Most often, the limiting component is phosphorus.

Since the second half of the XX century, not only natural factors have played a significant role in the geochemistry of phosphorus, but also industrial human activity. Phosphorus inflows from anthropogenic sources have led to eutrophication, not only of continental bodies of water but also of the seas and oceans [1].

One of the few tools enabling the study of quantitative parameters of phosphorus biodynamics in seawater is the short-lived phosphorus isotopes [3,4,5] of cosmogenic origin, ^32^P (T_1/2_ = 25.4 days) and ^33^P (T_1/2_ = 14.3 days) [6]. These isotopes are constantly forming during the interaction of cosmic rays with atmospheric argon and enter the World Ocean mainly with rainfall.

Some aspects of the phosphorus cycle in the Black Sea are poorly studied. Namely, the values of the parameters of time, rate, degree of circulation of ^33^P to inorganic and organic phosphate, and concentrations of various chemical forms of organic phosphorus are practically unknown. These aspects are important because the concentration of reduced forms of phosphorus (phosphonates and phosphinates) can be significant in the reducing environment of the hydrogen sulfide layer. Recent studies [7,8] indicate that these forms may play a key role in the biogeochemical cycle of phosphorus in the ocean.

It is necessary to know the volume of ^32^P and ^33^P arrivals with atmospheric precipitation to estimate their distribution in the ocean. More than 60 years ago, articles [9,10] showed that rainwater contained short-lived phosphorus isotopes ^32^P and ^33^P.

Even more significant is the role that ^32^P and ^33^P play in marine research as tracers for the study of biodynamics in the surface layer [3,11]. There have long been no methods to concentrate ^32^P and ^33^P because their concentration in seawater is three orders of magnitude lower than their concentration in wet atmospheric deposition. D. Lal first did this in his innovative works [12,13,14,15].

Later, more advanced methods for separating and concentrating ^32^P and ^33^P were proposed. Waser [16,17,18,19] describes the separation of ^32^P and ^33^P from rainwater samples on aluminum oxide. Doubled precipitation of ammonium phosphomolybdate (NH_4_)_3_[PMo_12_O_40_]∙2H_2_O and ammonium-magnesium phosphate NH_4_MgPO_4_∙6H_2_O is used for additional purification. Impurities are separated on a cation exchanger and anion exchanger to produce a counting sample for β-radiometry as NH_4_MgPO_4_∙6H_2_O.

While Benitez–Nelson [11,20,21] employed similar techniques, sorption of ^32^P and ^33^P from seawater was carried out on polypropylene cartridges impregnated with iron hydroxide (+3). A liquid sample was used in liquid scintillation spectrometry after purification using precipitation techniques, ion exchange resins, evaporation of the sample, and dissolution of the solid residue. Works [22,23] used this methodology as their foundation.

There have not been many studies in this field, and the existing published papers in Russian science are relatively new. Expeditionary work in the Laspi Bay was covered in earlier articles [24,25]. During these experiments, the ^32^P and ^33^P isotopes were concentrated from seawater using granulated aluminum oxide. However, it is important to carry out more research on how ^32^P and ^33^P affect the geochemical cycles of the most significant biogenic elements, particularly phosphorus, in the light of the anthropogenic load in the Sevastopol region. The objective of the study is to determine the variables that best describe the biodynamics of phosphorus, including the degree, rate, and time of ^33^P’s circulation to inorganic and organic phosphate in the Black Sea, as well as the most effective methods for the sorption and measurement of ^32^P and ^33^P.

This article is a logical follow-up to papers [26,27], which introduced and thoroughly characterized sorbents based on acrylate fiber and iron(+3) hydroxide obtained by various methods: Fe-NH—using non-hydrolyzed PAN (Polyacrylonitrile) and precipitation of Fe(OH)_3_ by ammonia [28]; Fe-SF—using ready-made Na_2_FeO_4_; Fe-H—using pre hydrolyzed PAN with precipitation of Fe(OH)_3_ by ammonia [29]. This article presents the evaluation of the effectiveness of ^32^P and ^33^P recovery from seawater by various sorbents as well as the seasonal variation of phosphorus biodynamics in the coastal region of Balaklava.

## 2. Materials and Methods

### 2.1. Materials

Seawater samples were filtered using nitrocellulose membrane filters for separation of suspended solids with pore size 0.45 µm and diameter 47 mm (JSC “Vladisart,” Vladimir, Russia), as well as polypropylene cartridges with pore sizes 0.5 µm FCPS1M series (Aquafilter Europe Ltd., Lodz, Poland). Active aluminum oxide (Technical Conditions 2163-002-25074287-2013) made by NPP “Tekhproekt” (Yekaterinburg, Russia), as well as cation exchanger KU-2-8 (State Standard 20298-74) and anion exchanger AB-17-8 (State Standard 20301-74) from (LLC “ReaChem”, Moscow, Russia) were used. Nitric acid, hydrochloric acid, ammonia, hydrogen peroxide, potassium dihydro orthophosphate, ammonium molybdate, ammonium chloride, and magnesium chloride (AO ReaKhim, Moscow, Russia) were qualified as pure for analysis (analytical grade).

### 2.2. The Method of Obtaining Sorbents

Papers [26,27] provided a detailed description of the method for creating sorbents based on PAN fibers and iron hydroxide (+3).

### 2.3. Sampling Methodology

**Figure 1:**
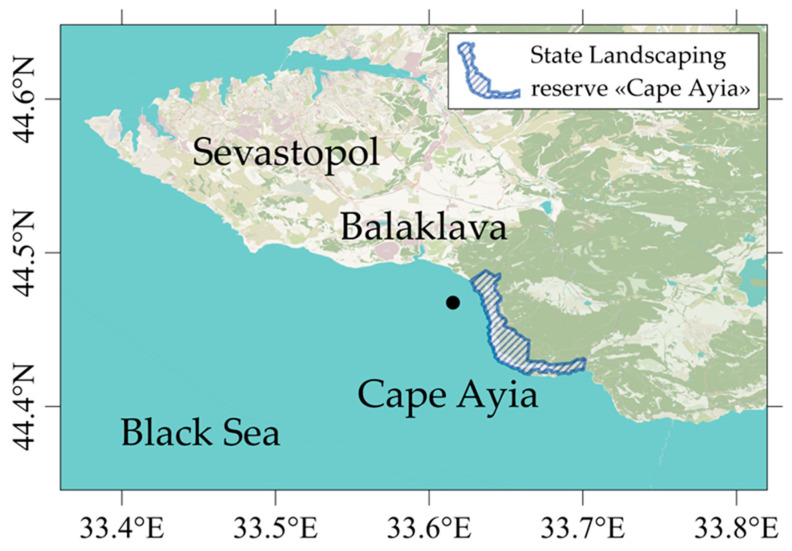
Sampling location in the Balaklava Coast area.

Coastal expeditions were conducted in the Balaklava coastal area (Figure 1) on 6 December 2021, 23 April, 10 July, and 8 October 2022, to study phosphorus biodynamics using ^32^P and ^33^P in different seasons. The sampling locations coordinates were 44.467486, 33.615426.

Seawater was pumped into plastic containers on board the ship from varying depths (3, 10, 20, and 30 m) using the submersible vibration pump UNIPUMP BAVLENETS BV 0,12-40-U5 (LLC “Sabline Service,” Moscow, Russia).

Before the sorption tests, samples were filtered through polypropylene cartridges with a 0.5 µm pore size from the FCPS1M series (Aquafilter Europe Ltd., Lodz, Poland). The prefilters were then ashed and used to determine the activity of radionuclides in the particulate form.

To collect seawater samples and determine the concentration of various forms of phosphorus, 125-mL polyethylene jars were specially made, washed with diluted hydrochloric acid and distilled water, and rinsed twice with the sampled water.

### 2.4. Determination of Hydrological Parameters

Hydrological parameters (salinity, temperature, and depth) were assessed using CTD probe “Hydrometrica 505-UTP” (LLC “Planeta Info”, St. Petersburg, Russia). Measurement errors are: temperature ± 0.05 °C, salinity ± 0.01‰, depth ± 0.04 m.

### 2.5. Determination of the Concentration of Various Forms of Phosphorus

Seawater samples were collected, filtered through membrane filters with pores 0.45 µm and 47 mm in diameter (JSC “Vladisart,” Vladimir, Russia), and then analyzed on the same day to determine the concentrations of different forms of phosphorus.

Concentrations of dissolved inorganic phosphorus (DIP, µmol/L) and total dissolved phosphorus (TDP, µmol/L) were measured using the accepted methods [30]. The difference between TDP and DIP was used to calculate the amount of dissolved organic phosphorus (DOP, µmol/L). The increase in filter mass, which was related to the amount of water pumped through them, was used to calculate the concentration of particulate matter.

The relative error of determination was 2% for DIP and TDP concentrations up to 2 µmol/L, and 1.5% for the concentration range of 2–8 µmol/L.

### 2.6. Sorption Concentration ^32^P and ^33^P

A single-column technique was used for sorption of ^32^P and ^33^P. Filtered seawater samples of 250 L or 1000 L were passed through a portion of sorbent of 25 mL or 200 mL using a LongerPump WT600-2J peristaltic pump (Longer Precision Pump Co., Baoding, China).

An aliquot of KH_2_PO_4_ solution was added to the seawater sample as a tracer until the level of phosphorus in the seawater reached 1 µmol/L to assess the yield. Every 10 to 50 L, the passed seawater was collected during the sorption process and placed in plastic tubes for further yield estimation.

A formula based on the initial and obtained concentrations was used to calculate the sorption efficiency of mineral phosphorus from seawater, which is equal to the sorption efficiency of ^32^P and ^33^P isotopes:

(1) $$R=\frac{C_{0}-C}{C_{0}}\cdot 100\%,$$

where *C*_0_ is the initial concentration of phosphate ion in seawater, μmol/L; *C* is the concentration of phosphate ion in the sample after the sorption, μmol/L.

### 2.7. Determination of the Specific Activity of ^32^P and ^33^P by Alpha-Beta Spectrometry with Radiochemical Preparation

Similar to the radiochemical preparation described in [22,23], precipitation techniques from classical analytical chemistry [31] were used.

Polypropylene cartridges (mechanical filters) were ashed in a SNOL-30/1300-I1p muffle furnace (AB UMEGA-GROUP, Utena, Lithuania) at 600 °C for 4 h. The ash was dissolved in 50 mL of concentrated nitric acid and 50 mL of 30% hydrogen peroxide to convert phosphorus into a dissolved form. To calculate the yield of stable phosphorus, an aliquot was taken.

After phosphorus sorption, PAN-Fe(OH)_3_ sorbents were ozolized in a SNOL-30/1300-I1p muffle furnace (AB UMEGA-GROUP, Utena, Lithuania) at 600 °C for 4 h. The ash was dissolved in 50 mL of 30% hydrogen peroxide and 50 mL of concentrated hydrochloric acid to convert phosphorus into a dissolved form.

The solution was separated by filtration. Then, 190 mL of concentrated nitric acid and 100 mL of concentrated ammonia were added to the solution. Then, 800 mL of distilled water were added, the solution was boiled, 6 mg of stable phosphorus were added, and potassium dihydro orthophosphate was added to determine the yield. To precipitate ammonium phosphomolybdate (NH_4_)_3_[PMo_12_O_40_]∙2H_2_O, 15 mL of a 15% ammonium molybdate solution was added:

KH_2_PO_4_ + 23HNO_3_ + 12(NH_4_)_2_MoO_4_ →  → (NH_4_)_3_[PMo_12_O_40_]∙2H_2_O + 21NH_4_NO_3_ + 2KNO_3_ + 10H_2_O. (2)

The precipitate was then washed with 50 mL of a 1 mol/L solution of nitric acid. To reprecipitate (NH_4_)_3_[PMo_12_O_40_]∙2H_2_O, it was dissolved in 20 mL of concentrated ammonia, and 50 mL of distilled water were added:

(NH_4_)_3_PMo_12_O_40_ + 21NH_4_OH → H_3_PO_4_ + 12(NH_4_)_2_MoO_4_ + 9H_2_O (3)

The resulting solution was filtered, and 30 mL of concentrated nitric acid were added and brought to boil. Then, 15 mL of a 15% ammonium molybdate solution were added dropwise, accompanied by vigorous stirring, and allowed to stand for 20 min:

H_3_PO_4_ + 21HNO_3_ + 12(NH_4_)_2_MoO_4_ →  → (NH_4_)_3_[PMo_12_O_40_]∙2H_2_O + 21NH_4_NO_3_ + 10H_2_O (4)

The solution was filtered off and then washed with 50 mL of a 1 mol/L solution of nitric acid. Then, 20 mL of concentrated ammonia were used to dissolve (NH_4_)_3_[PMo_12_O_40_]∙2H_2_O similar to the reaction Equation (3), and about 30 mL of concentrated hydrochloric acid were used to bring the pH value down to 7.

NH_4_MgPO_4_∙6H_2_O was precipitated. In an ice bath, 2 mL of concentrated ammonia and 40 mL of magnesia were added to the solution:

H_3_PO_4_ + MgCl_2_ + NH_4_Cl + 6H_2_O → NH_4_MgPO_4_∙6H_2_O + 3HCl (5)

The precipitate of NH_4_MgPO_4_∙6H_2_O was filtered off, washed with 0.5 mol/L ammonia, and then dissolved in 40 mL of a solution of 9 mol/L hydrochloric acid:

NH_4_MgPO_4_∙6H_2_O + 3HCl → MgCl_2_ + NH_4_Cl + H_3_PO_4_ + 6H_2_O (6)

The washed cation exchanger KU-2-8, or analog, was used to filter the solution. In addition, 10 mL of a 9 mol/L hydrochloric acid solution were used to wash the cation exchanger. Then, the washed anion exchanger AB-17-8, or an analog, was used to filter the solution. The anion exchanger was additionally washed with 10 mL of 9 mol/L hydrochloric acid solution.

The filtrate was dried by evaporation, dissolved in 3 mL of distilled water, and, if necessary, neutralized with concentrated ammonia before filtration using a cotton swab and a syringe. The residue was increased to 3 mL. An aliquoty of 50 µL was taken to determine the chemical yield.

The yield from the radiochemical preparation processes was determined for stable phosphorus as:

(7) $$\eta =\frac{m\left(P\right)}{m_{0}\left(P\right)}\cdot 100\%,$$

where *m*_0_(*P*) is the mass of applied stable phosphorus (6 mg) and phosphorus in the counting sample. For the sample, 15 mL of scintillation cocktail were added to 3 mL. In the entire channel range, ^32^P and ^33^P were measured on a Wallac 1220 Quantilus ultralow background spectrometer (Perkin Elmer, Turku, Finland) for at least 300 min. For ^32^P and ^33^P (*E_max_* > 156 keV), the counting efficiency is usually higher than 95% [22], and the uncertainty does not exceed 10%.

The volumetric activity of ^33^P was calculated as:

(8) $$A\left({}^{33}P\right)=\frac{R_{150-450}-R_{ph150-450}}{R\cdot \eta \cdot (1-\varphi )\cdot e^{-\lambda_{33}\cdot t}\cdot V}\;\mathrm{dpm}/m^{3},$$

where *R*_150–450_ is the channel count rate 150–450; *R_ph_*_150–450_ is the background count rate by channel 150–450; *η* is the chemical output; (*1 − φ*) is the portion of the sample taken on the liquid scintillation counting; *t* is the time elapsed from sampling to measurement; *λ*_33_ is the decay constant ^33^P, day^−1^ (0.0274 day^−1^); *R* is the degree of phosphorus recovery from seawater; and *V* is the sample volume, m^3^.

The volumetric activity of ^32^P was calculated as:

(9) $$A\left({}^{32}P\right)=\frac{R_{450-800}-R_{ph450-800}}{R\cdot \eta \cdot (1-\varphi )\cdot e^{-\lambda_{32}\cdot t}\cdot V}\;\mathrm{dpm}/m^{3},$$

where *R*_450–800_ is the channel count rate 450–800; *R_ph_*_450–800_ is the background count rate by channel 450–800; *λ*_32_ is the decay constant ^32^P, day^−1^ (0.04847 day^−1^).

### 2.8. Calculation of Quantitative Characteristics of Phosphorus Biodynamics

The circulation time of phosphorus to inorganic and particulate form was calculated as [13]:

(10) $$t=\frac{\ln\frac{R_{p}}{R_{s}}}{\lambda_{32}-\lambda_{33}}\;\mathrm{day},$$

where *t* is the circulation time (age of phosphorus); *R_p_*/*R_s_* is the ^33^P/^32^P activity ratio in the object and source of entry; and λ_32_ and λ_33_ are the radioactive decay constants of ^32^P and ^33^P isotopes.

The circulation rate of phosphorus in dissolved inorganic and particulate organic forms was calculated as [32]:

(11) $$\upsilon =\frac{c}{t}\;µ\mathrm{mol}/m^{3}\cdot \mathrm{day},$$

where *c* is the average concentration of phosphorus form; *t* is the phosphorus circulation time.

The circulation degree of phosphorus to dissolved inorganic and particulate organic forms was calculated as [32]:

(12) $$r=\frac{c}{t}\cdot h\;µ\mathrm{mol}/m^{2}\cdot \mathrm{day},$$

where *h* is the considered layer’s depth.

## 3. Results and Discussion

### 3.1. Assessing the Performance of Sorbents

**Figure 2:**
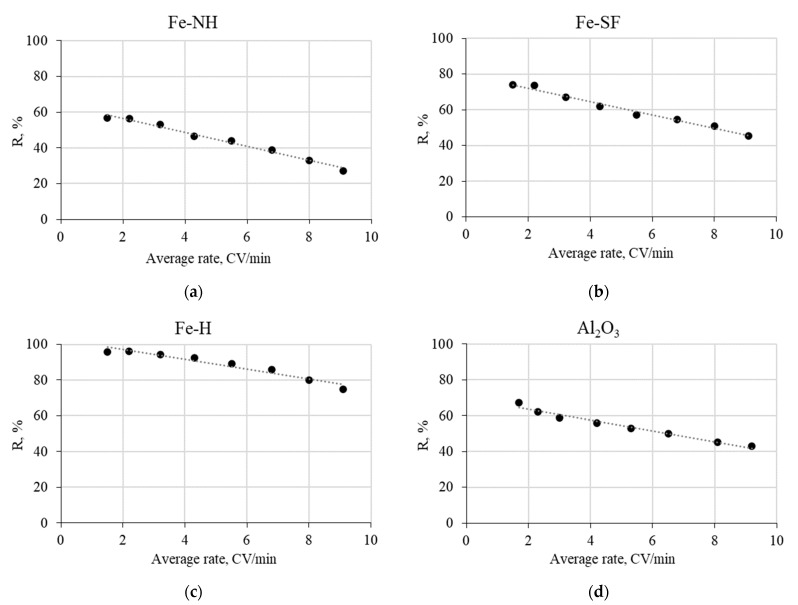
Dependence of ^32^P, ^33^P recovery efficiency (*R*, %) on the seawater flow rate by the sorbents: (**a**) Fe-NH; (**b**) Fe-SF; (**c**) Fe-H, (**d**) Al_2_O_3_ (single-column method, the volume of the sorbent 50 mL, volume of seawater—250 L).

Figure 2 illustrates the influence of seawater flow rate on the effectiveness of ^32^P and ^33^P recovery by a single-column method using granulated Al_2_O_3_ and fibrous sorbents PAN-Fe(OH)_3_ obtained by various methods.

These results showed that the highest efficiency of phosphorus recovery can be reached using Fe-H sorbent with a seawater flow rate of one-to-four column volumes per minute (CV/min).

### 3.2. Methodology for Recovery of ^32^P and ^33^P from Seawater

**Figure 3:**
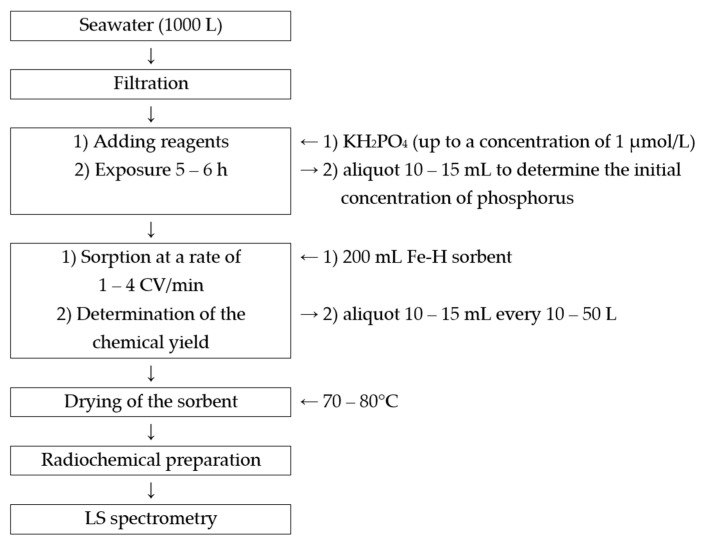
The developed methodology for ^32^P and ^33^P recovery from seawater.

Based on the findings, a methodology for the recovery of ^32^P and ^33^P from seawater was suggested (Figure 3):

1. Seawater (1000 L) is collected into a tank on board the ship and then filtered through a polypropylene filter with a 0.5 µm pore size. Large sample volumes are necessary because of low activity values of ^32^P and ^33^P in seawater of 1 to 5 dpm/m^3^ [11,23] and short half-lives of 14.27 and 25.35 days, respectively [6].
2. To evaluate the efficacy of the recovery process, a sample of potassium dihydro orthophosphate is added to the sampled seawater at a concentration of 1 µmol/L of phosphorus and left for 5–6 h to equalize the phosphorus concentration throughout the vessel.
3. Fe–H sorbent (200 mL, depending on the volume of seawater) is loaded onto the column.
4. Prepared seawater (1000 L) is passed through the sorbent-filled column at a rate of 1 to 4 CV/min.
5. To assess the recovery effectiveness of stable phosphorus, a sample of the passed seawater is taken periodically (every 10 to 50 L).
6. After sorption, the sorbent is dried in a desiccator at 70–80 °C.
7. ^32^P and ^33^P activity is determined by alpha-beta spectrometry method with radiochemical preparation according to the procedure described above.

Further studies on the concentration of ^32^P and ^33^P from seawater were performed according to the developed methodology.

### 3.3. Seasonal Variability of Phosphorus Biodynamics in the Balaklava Coastal Area

Coastal expeditions took place to the Balaklava coastal area on 6 December 2021, 23 April, 10 July, and 8 October 2022. The concentration of dissolved inorganic phosphorus and total dissolved phosphorus was measured, and three samples of 1000 L for ^32^P and ^33^P were taken.

#### 3.3.1. Concentrations of Different Forms of Phosphorus and Isotopes ^32^P and ^33^P

Table 1 displays the measured concentrations of different forms of phosphorus. They show the homogeneous change that is typical for the season and sampling location (surface layer, coastal area). The concentration of total particulate phosphorus, as well as the concentration of particulate matter, decreases with depth. At the same time, high values of particulate matter concentration (more than 1 mg/L) were noted, which is explained by the relative proximity of Balaklava sewage discharge into the open sea (more than 3 million m^3^/year) and known sources of submarine groundwater discharge.

**Figure 4:**
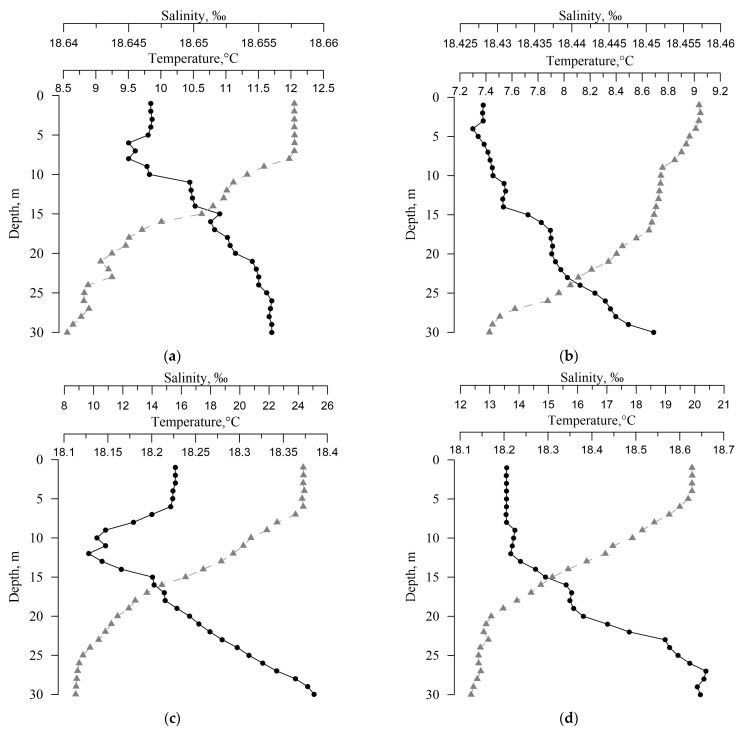
Salinity and temperature of seawater at different horizons: (**a**) winter 2021; (**b**) spring 2022; (**c**) summer 2022, (**d**) autumn 2022 (salinity (●), temperature (▲)).

Figure 4 shows the hydrological parameters (temperature and salinity) of the sampling stations in the Balaklava coastal area.

**Figure 5:**
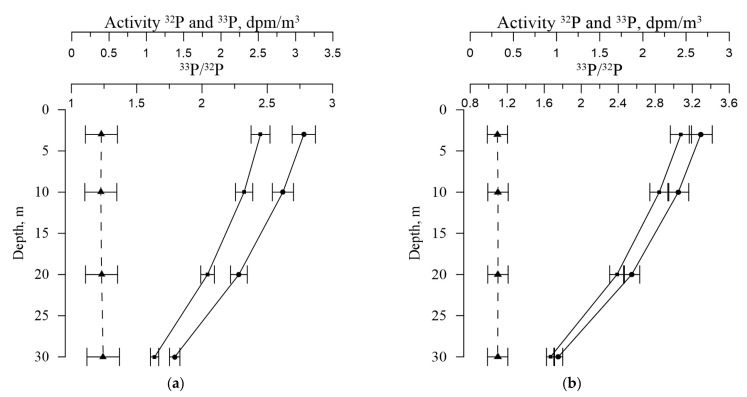

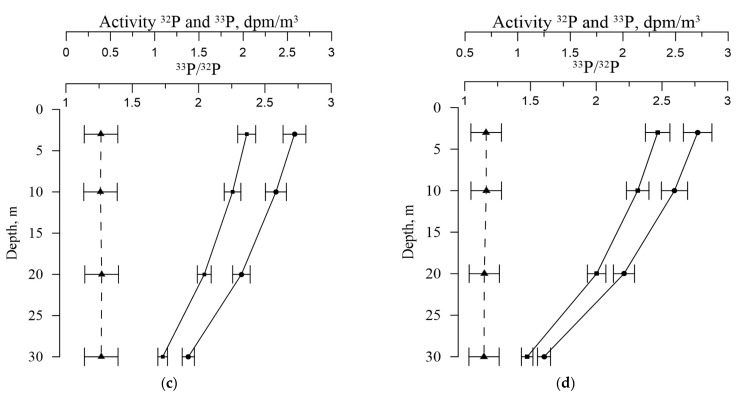
Values of ^32^P and ^33^P activity and ^33^P/^32^P ratio in seawater for different horizons: (**a**) winter 2021; (**b**) spring 2022; (**c**) summer 2022, (**d**) autumn 2022 (^32^P (■), ^33^P (●), ^33^P/^32^P ratio (▲)).

**Figure 6:**
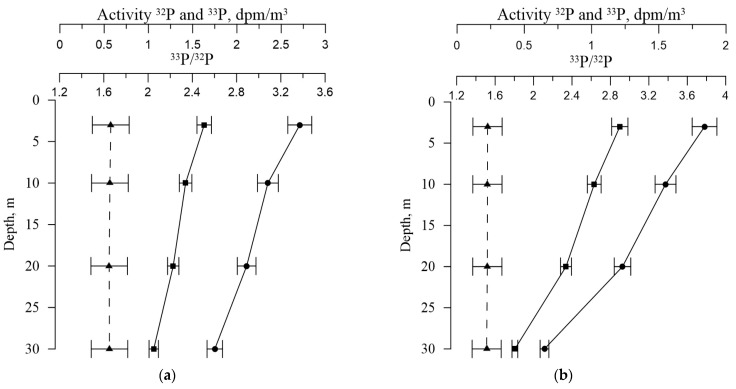

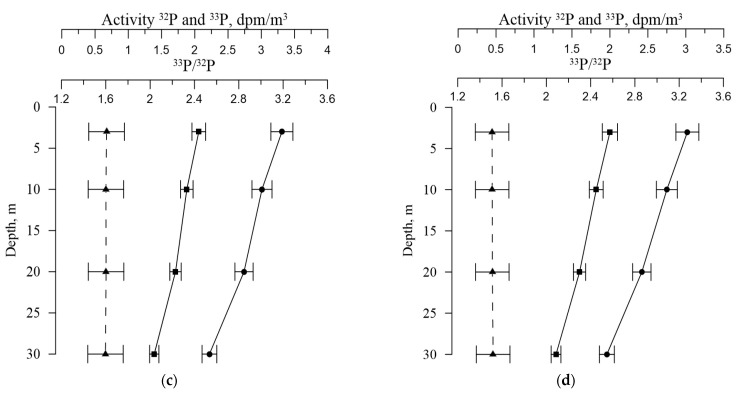
Values of ^32^P and ^33^P activity and ^33^P/^32^P ratio in the particulate matter for different horizons: (**a**) winter 2021; (**b**) spring 2022; (**c**) summer 2022, (**d**) autumn 2022 (^32^P (■), ^33^P (●), ^33^P/^32^P ratio (▲)).

Table 2 and Figure 5 and Figure 6 show the values of the volumetric activity of the isotopes ^32^P and ^33^P in dissolved and particulate forms, as well as the ^33^P/^32^P ratio for various horizons of seawater sampling. In both their dissolved and particulate forms, ^32^P and ^33^P activities are consistent with the data from the literature [21].

**Figure 7:**
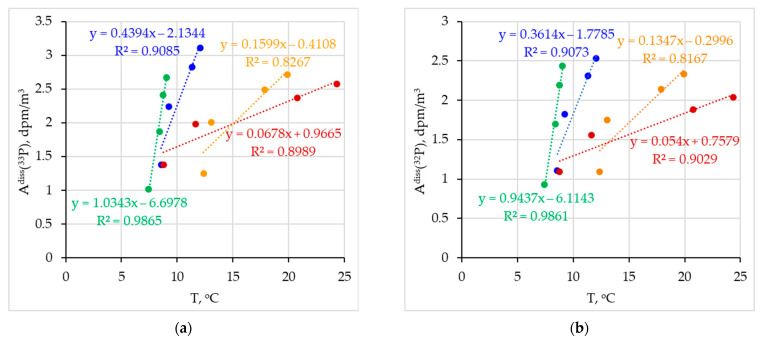

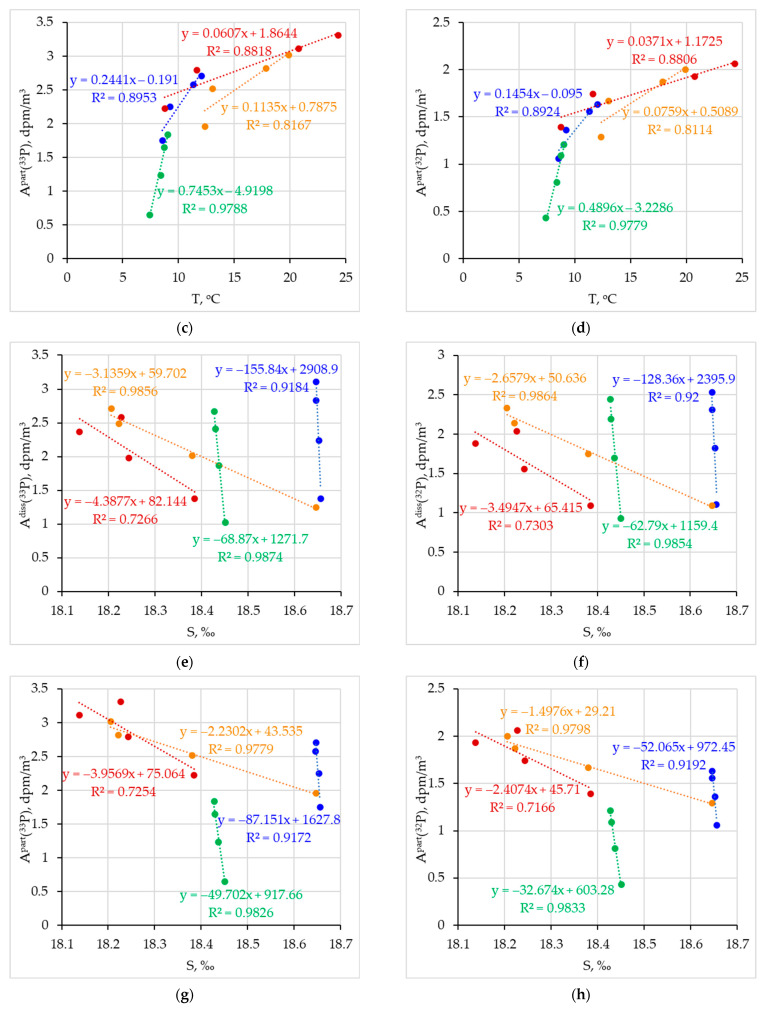
Dependences of ^33^P (**a**,**c**,**e**,**g**) and ^32^P (**b**,**d**,**f**,**h**) volumetric activity in dissolved (**a**,**b**,**e**,**f**) and particulate (**c**,**d**,**g**,**h**) form on temperature (**a**–**d**) and salinity (**e**–**h**) (winter 2021 (●), spring 2022 (●), summer 2022 (●), autumn 2022(●)).

Figure 7 shows the dependences of the activities of ^33^P and ^32^P in dissolved and particulate form on temperature and salinity. Smaller correlations with salinity and temperature are observed in summer and autumn due to two factors. The first is stable stratification due to a large temperature gradient, and the second is the active involvement of phosphorus in biochemical processes during these seasons. In winter and spring, due to the destruction of stratification, intensive mixing (which can be seen in Figure 4) and the slowdown in the processes of consumption and release of phosphorus, its dependence on temperature and salinity is not so significant and correlates well with them, mainly due to the fact that the content of ^32^P and ^33^P is determined by their short half-lives.

#### 3.3.2. Parameters of Phosphorus Biodynamics

The values of circulation time, circulation rate, and circulation degree in dissolved inorganic form were calculated using formulas (5–7), respectively, based on the ^33^P/^32^P ratio in seawater at different horizons and in the source-atmospheric deposition (1.12 in December 2021, 0.99 in April 2022, 1.20 in July 2022, and 1.08 in October 2022). Formulas (5)–(7) were used to calculate the circulation time, rate, and degree in the dissolved organic form from the values of ^33^P/^32^P in the suspension and the source seawater (Table 3). The outcomes are consistent with the figures from [21].

The minimum and maximum values of the phosphorus circulation period were observed in the warmest months (July and October), and the correlation between the two can be seen. The minimum values were observed in the coldest months: December and April.

An analysis of the obtained parameters of phosphorus biodynamics, namely, the circulation rate and degree in spring and summer, shows increased values in comparison with the parameters obtained in winter and autumn. Thus, in spring, despite the lower water temperature, the parameters of phosphorus biodynamics are higher. In our opinion, this is due to increased surface runoff due to the abundance of rainfall from March to April 2022. As a result, not only phosphorus of terrigenous origin enters the marine environment, but also of anthropogenic origin (domestic and agricultural effluents). In particular, the first application of fertilizers to the abundance of Balaklava vineyards occurs from the end of March to the beginning of April, which undoubtedly affects the parameters of biodynamics.

The increase in parameters in the summer is explained by the resort activities in Balaklava, which negatively affects the state of the marine ecosystem.

Thus, despite the obtained typical concentrations of various forms of phosphorus (Table 1), the parameters of biodynamics give a more complete picture of the anthropogenic load on the region under study.

## 4. Conclusions

This study determines the effectiveness of various sorbents to recover phosphorus isotopes from seawater. The results showed that using the Fe–H sorbent at a seawater flow rate of 1 to 4 CV/min results in the highest efficiency of phosphorus recovery.

A method for removing ^32^P and ^33^P isotopes from seawater using Fe–H sorbent has been developed. The technique used a single-column method with the addition of micro quantities of potassium dihydro phosphate as a tracer of recovery efficiency.

For various seawater sampling horizons, the volumetric activities of ^32^P and ^33^P in dissolved and particulate forms, as well as the ^33^P/^32^P ratio, were assessed.

In the region of the Balaklava coast, the seasonal variability of the phosphorus biodynamics parameters has been identified. Calculations were made regarding the amount of phosphorus converted to inorganic and particulate organic forms over time and rate. The duration of phosphorus turnover correlated with water temperature; the minimum values of the turnover period were noted in the warmest months (July and October), and the maximum in the colder months (December and April).

Elevated values of parameters of phosphorus biodynamics in the spring and summer were determined, which can be explained by the peculiarity of the economic and resort activities of Balaklava that negatively affect the state of the marine ecosystem.

Since the tests were carried out near the Cape Aya State Natural Landscape Reserve of regional significance, it is possible to use the results of this work for the development of an integral system of specially protected natural areas and to maintain the ecological balance, which is one of the goals of the reserve.

## Figures and Tables

**Table 1:** Concentration values for different forms of phosphorus in different seasons.

Date	Depth, m	Concentration Values for Various Forms of Phosphorus, µmol/L				Concentration Particulate Matter, mg/L
		DIP	TDP	DOP	TSP	
6 December 2021	3	0.08	0.21	0.13	0.11	1.94
	10	0.07	0.20	0.13	0.09	1.61
	20	0.07	0.21	0.14	0.07	1.27
	30	0.07	0.21	0.14	0.07	0.96
23 April 2022	3	0.12	0.23	0.11	0.12	2.12
	10	0.11	0.23	0.12	0.10	1.76
	20	0.11	0.21	0.10	0.09	1.35
	30	0.10	0.22	0.12	0.07	1.05
10 July 2022	3	0.06	0.17	0.11	0.08	1.45
	10	0.06	0.16	0.10	0.07	1.51
	20	0.05	0.16	0.11	0.07	1.19
	30	0.05	0.15	0.10	0.06	0.89
8 October 2022	3	0.05	0.25	0.20	0.07	1.51
	10	0.05	0.24	0.19	0.06	1.56
	20	0.04	0.22	0.18	0.04	1.32
	30	0.03	0.22	0.19	0.04	1.01

**Table 2:** Values of ^32^P and ^33^P activity and the ratio ^33^P/^32^P in different seasons.

Date	Depth, m	Dissolved Form			Particulate Form		
		A(^33^P), dpm/m^3^	A(^32^P), dpm/m^3^	^33^P/^32^P	A(^33^P), dpm/m^3^	A(^32^P), dpm/m^3^	^33^P/^32^P
6 December 2021	3	3.11 ± 0.16	2.53 ± 0.13	1.23 ± 0.12	2.71 ± 0.14	1.63 ± 0.08	1.66 ± 0.17
	10	2.83 ± 0.14	2.31 ± 0.12	1.23 ± 0.12	2.35 ± 0.12	1.42 ± 0.07	1.65 ± 0.17
	20	2.24 ± 0.11	1.82 ± 0.09	1.23 ± 0.12	2.11 ± 0.11	1.28 ± 0.06	1.65 ± 0.17
	30	1.38 ± 0.07	1.11 ± 0.06	1.24 ± 0.12	1.75 ± 0.09	1.06 ± 0.05	1.65 ± 0.17
23 April 2022	3	2.67 ± 0.13	2.44 ± 0.12	1.09 ± 0.11	1.84 ± 0.09	1.21 ± 0.06	1.52 ± 0.15
	10	2.41 ± 0.12	2.19 ± 0.11	1.10 ± 0.11	1.55 ± 0.08	1.02 ± 0.05	1.52 ± 0.15
	20	1.87 ± 0.09	1.70 ± 0.09	1.10 ± 0.11	1.23 ± 0.06	0.81 ± 0.04	1.52 ± 0.15
	30	1.02 ± 0.05	0.93 ± 0.05	1.10 ± 0.11	0.65 ± 0.03	0.43 ± 0.02	1.51 ± 0.15
10 July 2022	3	2.58 ± 0.13	2.04 ± 0.10	1.26 ± 0.13	3.31 ± 0.17	2.06 ± 0.10	1.61 ± 0.16
	10	2.37 ± 0.12	1.88 ± 0.09	1.26 ± 0.13	3.01 ± 0.15	1.88 ± 0.09	1.60 ± 0.16
	20	1.98 ± 0.10	1.56 ± 0.08	1.27 ± 0.13	2.74 ± 0.14	1.71 ± 0.09	1.60 ± 0.16
	30	1.38 ± 0.07	1.09 ± 0.05	1.27 ± 0.13	2.22 ± 0.11	1.39 ± 0.07	1.60 ± 0.16
8 October 2022	3	2.71 ± 0.14	2.33 ± 0.12	1.16 ± 0.12	3.02 ± 0.15	2.00 ± 0.10	1.51 ± 0.15
	10	2.49 ± 0.12	2.14 ± 0.11	1.16 ± 0.12	2.75 ± 0.14	1.82 ± 0.09	1.51 ± 0.15
	20	2.01 ± 0.10	1.75 ± 0.09	1.15 ± 0.12	2.42 ± 0.12	1.60 ± 0.08	1.51 ± 0.15
	30	1.25 ± 0.06	1.09 ± 0.05	1.15 ± 0.12	1.96 ± 0.10	1.29 ± 0.06	1.52 ± 0.15

**Table 3:** Parameters of phosphorus biodynamics.

Date	Circulation Time *t_av_*, Day		Circulation Rate *υ_av_*, µmol/m^3^·Day		Circulation Degree *r_av_*, µmol/m^2^·Day	
	Dissolved Form	Particulate Form	Dissolved Form	Particulate Form	Dissolved Form	Particulate Form
6 December 2021	4.5	13.9	16.1	6.1	484	184
23 April 2022	4.9	15.2	22.6	6.2	678	187
10 July 2022	2.5	11.1	22.1	6.3	663	189
8 October 2022	3.2	12.7	13.4	4.1	401	124

## Data Availability

Not applicable.
